# Pharmacometabolomic Assessment of Metformin in Non-diabetic, African Americans

**DOI:** 10.3389/fphar.2016.00135

**Published:** 2016-06-14

**Authors:** Daniel M. Rotroff, Noffisat O. Oki, Xiaomin Liang, Sook Wah Yee, Sophie L. Stocker, Daniel G. Corum, Michele Meisner, Oliver Fiehn, Alison A. Motsinger-Reif, Kathleen M. Giacomini, Rima Kaddurah-Daouk

**Affiliations:** ^1^Bioinformatics Research Center, North Carolina State UniversityRaleigh, NC, USA; ^2^Department of Statistics, North Carolina State UniversityRaleigh, NC, USA; ^3^Department of Bioengineering and Therapeutic Sciences, University of California San FranciscoSan Francisco, CA, USA; ^4^Department of Regenerative Medicine and Cell Biology, Medical University of South CarolinaCharleston, SC, USA; ^5^UC Davis Genome Center, University of California DavisDavis, CA, USA; ^6^Department of Biochemistry, King Abdulaziz UniversityJeddah, Saudi-Arabia; ^7^Department of Psychiatry and Behavioral Sciences, Duke UniversityDurham, NC, USA; ^8^Duke Institute for Brain Sciences, Duke UniversityDurham, NC, USA

**Keywords:** pharmacometabolomics, metformin, metabolism, precision medicine

## Abstract

Millions of individuals are diagnosed with type 2 diabetes mellitus (T2D), which increases the risk for a plethora of adverse outcomes including cardiovascular events and kidney disease. Metformin is the most widely prescribed medication for the treatment of T2D; however, its mechanism is not fully understood and individuals vary in their response to this therapy. Here, we use a non-targeted, pharmacometabolomics approach to measure 384 metabolites in 33 non-diabetic, African American subjects dosed with metformin. Three plasma samples were obtained from each subject, one before and two after metformin administration. Validation studies were performed in wildtype mice given metformin. Fifty-four metabolites (including 21 unknowns) were significantly altered upon metformin administration, and 12 metabolites (including six unknowns) were significantly associated with metformin-induced change in glucose (*q* < 0.2). Of note, indole-3-acetate, a metabolite produced by gut microbes, and 4-hydroxyproline were modulated following metformin exposure in both humans and mice. 2-Hydroxybutanoic acid, a metabolite previously associated with insulin resistance and an early biomarker of T2D, was positively correlated with fasting glucose levels as well as glucose levels following oral glucose tolerance tests after metformin administration. Pathway analysis revealed that metformin administration was associated with changes in a number of metabolites in the urea cycle and in purine metabolic pathways (*q* < 0.01). Further research is needed to validate the biomarkers of metformin exposure and response identified in this study, and to understand the role of metformin in ammonia detoxification, protein degradation and purine metabolic pathways.

## Introduction

It is estimated that ~11% of people in the United States aged 20 years or older have diagnosed or undiagnosed type 2 diabetes mellitus (T2D), and 35% of adults in the same age group are estimated to have prediabetes based on fasting glucose or hemoblogin A1c levels (Centers for Disease Control and Prevention, 2014). Metformin is an effective and extensively prescribed medication for the control of T2D, and is now being investigated for its potential beneficial effects in the prevention or treatment of other diseases, such as a wide-ranging number of cancers (Evans et al., 2005; Zakikhani et al., 2006, 2008; Duncan and Schmidt, 2009). Although metformin has been in use as a glucose lowering drug for several decades, its underlying mechanism of action, as well as its effects on metabolism are not well understood. Metformin has been shown to impact the adenosine-5-phosphate kinase (AMPK) signaling pathway (Zhou et al., 2001), which is thought to be an important component in metformin's glucose-lowering mechanism. Other pathways, such as the mTOR pathway have also been implicated in the beneficial effects of metformin including its antiproliferative effects in cancer treatment (Sahra et al., 2011). Recently, Madiraju et al. (2014) showed that metformin reduces gluconeogenesis via inhibition of a mitochondrial enzyme, glycerophosphate dehydrogenase in the liver (Madiraju et al., 2014). Understanding the complex mechanisms of action of metformin is of particular interest as it may lead to validated biomarkers that can be used to identify individuals most likely to respond to the drug, as well as those most likely to experience adverse drug effects.

Metabolomics is a rapidly emerging field with the potential to transform our understanding of mechanisms of drug action and the molecular basis for variation in drug response by characterizing metabolism at an “omic” level (Kaddurah-Daouk et al., 2014, 2015). The “metabotype,” the metabolic “signature” of a patient, is a unique identity that contains information about drug response and disease heterogeneity. Metabolic signatures of drug exposure can now identify pathways involved in both drug efficacy and adverse drug reactions (Trupp et al., 2012; Kaddurah-Daouk et al., 2013; Lewis et al., 2013; Zhu et al., 2013; Rotroff et al., 2015). The application of metabolomics to study drug effects and variation in drug response is creating “pharmacometabolomics,” a discipline that complements pharmacogenomics and clinical pharmacology by capturing the metabolic signatures associated with drug exposure, therapeutic and adverse drug response as well as interindividual differences in these signatures.

Several studies have used metabolomic approaches to identify metabolites in plasma and urine that were associated with metformin exposure in non-diabetic subjects or patients with T2D of North East Asian (Cai et al., 2009; Huo et al., 2009; Song et al., 2012; Cho et al., 2015; Xu et al., 2015). In this study, we focused on a non-diabetic, African-American population, and used a pharmacometabolomic approach to gain further insights about the mechanisms of action of metformin. Our goals were to identify metabolic signatures associated with metformin exposure and its pharmacologic action on oral glucose tolerance. Using a non-targeted, GC-TOF mass spectrometry based metabolomics platform, we investigated the effect of metformin on a wide range of metabolites, and their relationship with changes in plasma glucose. Although this technology represents the state-of-the-art, many metabolites have not been previously annotated, highlighting the vast potential for expanding our understanding of novel biology, A pathway enrichment approach was used to gain novel insights to the biological pathways impacted by metformin treatment. Finally we performed a follow-up study in mice to determine if the most significantly affected metabolites from the clinical study were replicated in mice exposed to metformin.

## Materials and methods

### Subject recruitment and study design

Subjects were recruited directly from the Study of Pharmacogenetics in Ethnically Diverse Populations (IRB 10-03167). Thirty-three subjects, who were of African-American ethnicity, were enrolled in this study. Males and females between the ages of 18 and 45 were included. Screening included a comprehensive medical history, physical examination, and laboratory studies (complete blood count, electrolytes, blood urea nitrogen and creatinine, albumin, and liver enzymes). Subjects of an ethnic background other than African-American, women who were pregnant, people who were not between the ages of 18 and 45 years, and individuals with certain health conditions were excluded (e.g., elevated liver enzymes, anemia, and elevated creatinine concentrations). This study has been described previously (Stocker et al., 2013). Subjects did not have a diagnosis of diabetes and their laboratory values were in the normal range.

Subjects were asked to maintain stable physical activity levels for 7 days before starting the study. Individuals met with a dietitian to establish a 3-day meal plan that maintained carbohydrate intake at 200–250 g per day before being admitted to the General Clinical Research Center at San Francisco General Hospital for 3 days (72 h). At 7:00 p.m. on day 0, subjects were admitted to the General Clinical Research Center at San Francisco General Hospital for overnight fasting (10 h). At 8:00 a.m. on day 1, a 3-h oral glucose tolerance test (OGTT) was administered (75 g glucose solution). In the evening on day 1 (7:00 p.m.), subjects were dosed orally with 1000 mg metformin (Major Pharmaceuticals, Livonia, MI), followed by a 10-h overnight fasting. On the next morning (7:30 a.m.; day 2), subjects received an additional oral dose of 850 mg of metformin, and a second OGTT was administered 2 h after this metformin administration. Throughout the study, standardized meals were provided. In addition, subjects were asked to drink 8 oz of water every 2 h to maintain urine flow and pH.

Blood samples were collected at various times before and after drug administration into “heparin (plasma separation) tube.” Metformin concentrations in the plasma were assayed by a validated liquid chromatography-tandem mass spectrometry method (Stocker et al., 2013). Glucose concentrations in plasma were determined using standard colorimetric assays. Pharmacokinetic metrics of metformin and glucose were calculated as described previously (Stocker et al., 2013).

**Figure 1:**
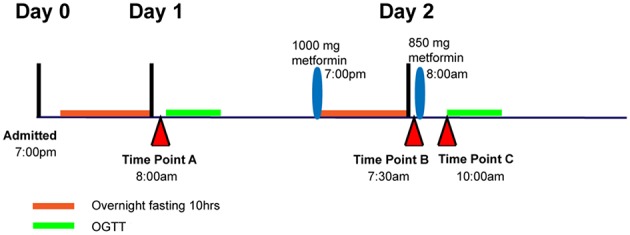
**Diagram showing time point A, B, and C relative to metformin administration and oral glucose tolerance tests, OGTT**.

Three plasma samples per individual were selected to measure metabolite levels using the GC-TOF platform at the West Coast Metabolomics Center at UC Davis. Time point A samples were taken at 8 a.m. on day 1 and represent the baseline plasma level after 10 h overnight fasting prior to dosing with metformin. Time point B samples were taken at 7:30 a.m. on day 2, which was 10 h of overnight fasting and 12.5 h after the first dose of metformin. Time point C samples were taken 2 h after the second dose of metformin and is referred to as the maximal plasma concentration of metformin (Figure 1).

### Metabolomic profiling

Using the GC-TOF platform, 384 metabolites were quantified, of which 155 have been structurally identified. This platform, samples preparations and methods for profiling have been described in several studies by the Pharmacometabolomic Research Network (Ji et al., 2011; Trupp et al., 2012; Wikoff et al., 2013; Zhu et al., 2013).

### Data analysis

#### Data processing

Metabolite data was normalized by performing a log(x+1) transformation and the population means were imputed for missing values. All analyses were conducted using the statistical software, R (R Development Core Team, 2014).

#### Signature of exposure of metabolites to metformin

The effect of metformin exposure on metabolite levels between time points: A (baseline pre-metformin day 1), B (baseline day 2), and C (peak of metformin day 2) were tested using paired Wilcoxon tests. Spearman's rank correlation was used to test for correlations between the significant metabolites and to determine the direction of association. Results were corrected for multiple testing using an FDR approach and results with *q* < 0.2 were considered to be statistically significant (Benjamini and Hochberg, 1995). Hierarchical clustering was performed on significant metabolites (*q* < 0.2) at the three time points using modulated modularity clustering and the Spearman's rank correlation (Stone and Ayroles, 2009).

#### Signature of response of metabolites to metformin concentration

Univariate association for each metabolite with metformin concentration in plasma at the three time points was determined using a linear regression model. The analysis was performed using two variations of response: (1) metformin AUC, and (2) peak (Cmax) metformin concentration. Gender, age, body mass index (BMI), weight, and height were tested for association with each response variable using a Pearson correlation coefficient = |r| > 0.15. BMI was the only covariate that met this criterion and was subsequently included in the model, with an *r* = −0.25 and −0.13 for Cmax and AUC, respectively. Additional information about the linear model and covariate selection are available in the Supplementary Material.

#### Correlations of metabolites with glucose change

Metabolites associations with changes in glucose upon metformin exposure were tested using the Spearman's correlation between glucose response and the metabolites at each time point separately (A, B, and C), and the metabolite changes for each time point (A to B, B to C, and A to C). Glucose response was defined as either the absolute difference of AUC glucose measurements pre- and post-metformin treatment, or the post-metformin AUC glucose measurement. Results were corrected for multiple comparisons using an FDR approach, and a threshold of *q* < 0.2 was used for statistical significance (Benjamini and Hochberg, 1995).

#### Pathway analysis

Two separate pathway analyses were conducted using either metabolites significantly different between the three time points (*q* < 0.2), and metabolites significantly associated with glucose change pre- and post-metformin (*q* < 0.2). Metabolite pathway data was obtained from the Human Metabolome Database (HMDB v3.5; Wishart et al., 2013), and metabolites in HMDB not attributed to a pathway and “unknown” metabolites in our data set were excluded from the pathway analysis. Pathways were tested for enrichment using an over-representation analysis (ORA) approach, where overlapping metabolites in each group and pathway were tested for statistical significance using the hypergeometric distribution. Finally, significance values were adjusted for multiple comparisons using an FDR approach (Benjamini and Hochberg, 1995).

#### Follow-up mouse study

Overlapping metabolites that were significant in the signature of metformin response analysis in subjects were tested to determine whether significant metabolites were replicated in a mouse model. Eighteen, 12-week old male C57BL/6J mice were randomly placed into three treatment groups of either saline, 50 mg/kg metformin, or 150 mg/kg metformin. Treatments were administered intraperitoneally each day for 7 days. Mice were fasted 16 h before blood sample and liver collection. The animal protocol was approved by UCSF IACUC (protocol number: AN119364). Frozen serum and liver samples were sent to the West Coast Metabolomics Center at UC Davis for metabolomic analysis using the GC-TOF platform. Metabolite data processing and analysis was conducted using the same methods as stated above for the human samples. Metabolite changes with multiple test corrected *q* < 0.3 were considered to have replicated.

## Results

### Signature of exposure to metformin from time points A to B

Metformin exposure significantly altered 17 metabolites between time points A (overnight fasting, pre-metformin) and B (overnight fasting, 12.5 h post-metformin first dose; *q* < 0.2), 9 of which have been structurally identified (Table 1). Compared to baseline, the five most significantly increased metabolites were 629905, 629906, 4-hydroxypoline, 781707, and 203221, and the five most significantly decreased metabolites were citrulline, ornithine, methionine sulfoxide, 300348, and 223618.

**Table 1:** **Signature of exposure to metformin**.

**Metabolite**	**Change**	***q*-value**
^*^629905	↑	0.000114
^*^629906	↑	0.000114
Citrulline	↓	0.00627
Ornithine	↓	0.008409
4-hydroxyproline	↑	0.037956
^*^781707	↑	0.077604
Methionine sulfoxide	↓	0.077604
^*^203221	↑	0.085495
^*^300348	↓	0.085495
^*^223618	↓	0.094211
^*^199203	↓	0.118784
Glutamic acid	↑	0.137676
Indole-3-acetate	↑	0.137676
Glutamine	↓	0.151364
Threonic acid	↓	0.151364
^*^223973	↑	0.16033
Xylulose NIST	↑	0.16033

*Significant metabolites that either increased or decreased between time points A (pre-metformin) and B (post-first dose of metformin) in non-diabetic African-American subjects (n = 33)*.

* *Metabolite compounds yet to be structurally identified*.

### Signature of exposure to metformin from time points B to C

Metformin exposure from time points B (overnight fasting, 12.5 h post-metformin first dose) and C (2 h post-metformin second dose) significantly altered 23 metabolites (*q* < 0.2), including 14 of which are structurally known (Table 2). Compared to time point B, the five most significantly increased metabolites were 629905, 629906, 300195, ribose, and 214535; while the five most significantly decreased metabolites were indole-3-acetate, levoglucosan, glycerol-3-galactoside, 2-deoxyerythritol NIST, and adenosine-5-phosphate. The two most significant metabolites (629905 and 629906) in this analysis were also the most significant metabolites identified when comparing time points A and B.

**Table 2:** **Signature of exposure to metformin**.

**Metabolite**	**Change**	***q*-value**
^*^629905	↑	0.000131
^*^629906	↑	0.004753
Indole-3-acetate	↓	0.004753
Levoglucosan	↓	0.00725
Glycerol-3-galactoside	↓	0.010948
2-deoxyerythritol NIST	↓	0.023427
Adenosine-5-phosphate	↓	0.023427
Butane-2,3-diol NIST	↓	0.023427
Inosine	↓	0.023427
^*^300195	↑	0.032767
Ribose	↑	0.032767
2-Hydroxyglutaric acid	↓	0.039829
^*^214535	↑	0.039829
^*^218765	↑	0.039829
^*^228377	↑	0.042798
Arachidonic acid	↑	0.046093
^*^238384	↑	0.062027
Lathosterol (NIST)	↓	0.062027
Maleimide	↑	0.062027
^*^748746	↑	0.062877
Glucuronic acid	↓	0.125629
^*^460930	↑	0.134797
Pyruvic acid	↓	0.153147

*Significant metabolites that either increased or decreased between time points B (post-first dose of metformin) and C (post-metformin second dose) in non-diabetic African-American subjects (n = 33)*.

* *Metabolite compounds yet to be structurally identified*.

### Signature of exposure to metformin from time points A to C

The most significant impact on metabolite concentrations, as expected, occurred between baseline (time point A, pre-metformin treatment) and the time at which metformin concentrations in plasma reached their maximum (time point C, 2 h post-metformin second dose, Cmax). A total of 38 metabolites were significantly changed between the two time intervals (*q* < 0.2; Table 3). Of the 38, 25 have been structurally identified. Compared to the pre-metformin treatment (baseline), time point A, the five most significantly increased metabolites were 629905, 228605, 629906, hypoxanthine, and maltose; whereas, the most significantly decreased metabolites were citrulline, tyrosine, ornithine, 223618, and 199203. The three most significant metabolites overall (629905, 228605, 629906) were all previously unknown. Furthermore, 629905 and 629906 were identified as significant metabolites in multiple signatures of metformin exposure analyses, as described above.

**Table 3:** **Signature of exposure to metformin**.

**Metabolite**	**Change**	***q*-value**
^*^629905	↑	8.19E-08
^*^228605	↑	5.04E-05
^*^629906	↑	5.04E-05
Citrulline	↓	5.04E-05
Hypoxanthine	↑	5.04E-05
Maltose	↑	5.04E-05
Ribose	↑	5.04E-05
Glutamic acid	↑	0.000979753
Tyrosine	↓	0.001993223
Cellobiotol	↑	0.007832535
Ornithine	↓	0.007832535
^*^223618	↓	0.029131013
^*^199203	↓	0.029829182
Glucuronic acid	↓	0.029829182
Maltotriose	↑	0.034537934
^*^284946	↓	0.038066794
4-hydroxyproline	↑	0.038066794
Inosine	↓	0.038066794
Glycerol-3-galactoside	↓	0.038621793
Xylulose NIST	↑	0.039272793
Uridine	↑	0.040013669
^*^322652	↑	0.040839767
Naproxen	↓	0.050799173
Cytidine-5′-diphosphate	↑	0.055347551
Hippuric acid	↑	0.060289124
^*^235327	↑	0.065649756
^*^223973	↑	0.071456533
^*^199786	↑	0.073205052
^*^353747	↓	0.095009578
Glutamine	↓	0.097307921
Levoglucosan	↓	0.105569426
^*^748746	↑	0.120340346
Aspartic acid	↑	0.120340346
Mannitol	↓	0.120340346
Arachidonic acid	↑	0.137600629
Butane-2,3-diol NIST	↓	0.14882581
Maleimide	↑	0.152636013
^*^300348	↓	0.173640266

*Significant metabolites that either increased or decreased between time points A (pre-metformin) and C (post-metformin second dose) in non-diabetic African-American subjects (n = 33)*.

* *Metabolite compounds yet to be structurally identified*.

**Figure 2:**
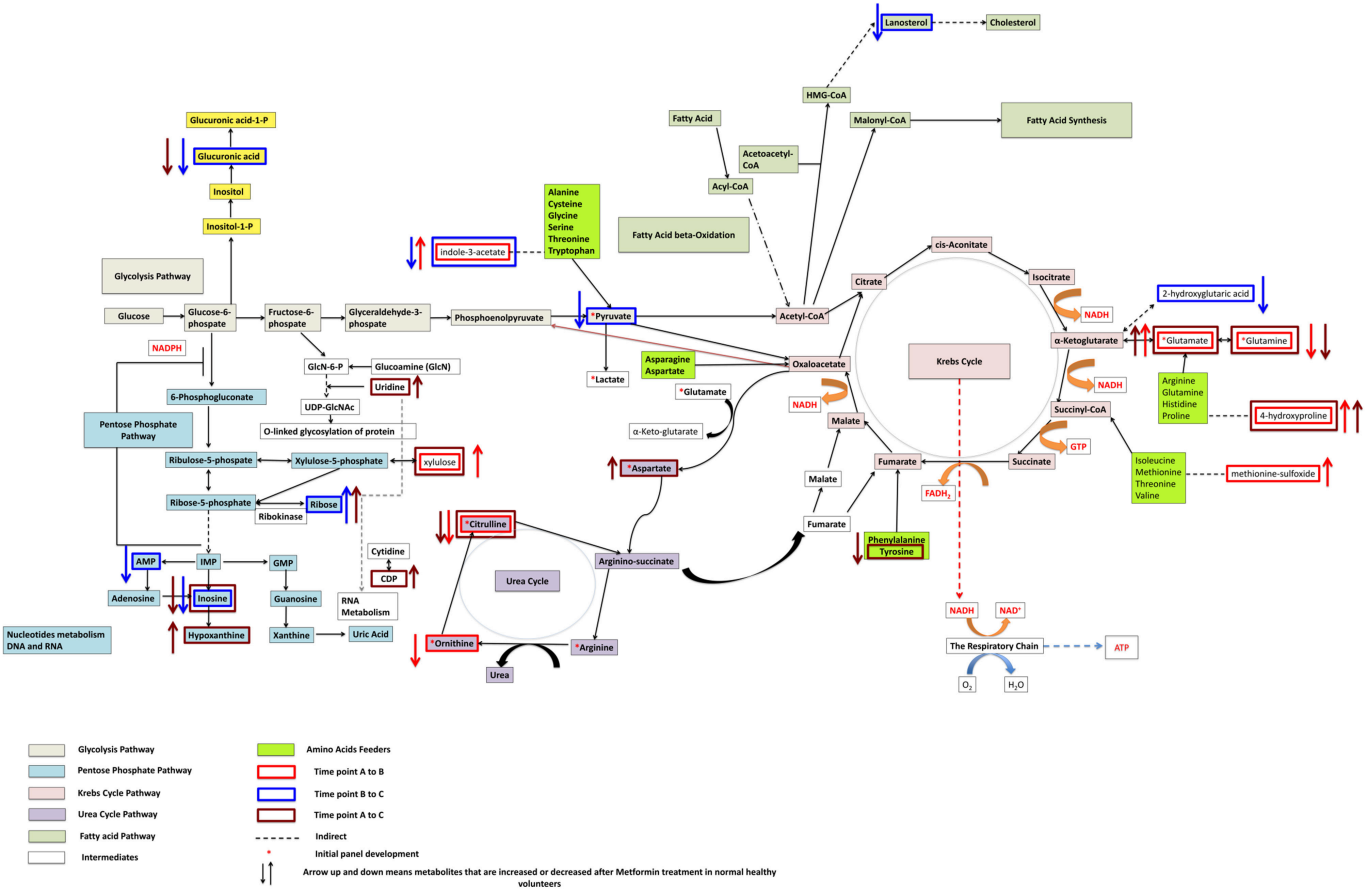
**Pathways significantly impacted following metformin administration and associated with metformin's effects on glucose levels after an oral glucose tolerance test in non-diabetic African-American subjects**.

Individual metabolite changes and their pathway context are shown in Figure 2. Overall, metabolites 629905 and 629906, were found to be significant when comparing all three different time points (A and B, B and C, A and C). Additional, spectral analysis suggests that these metabolites are likely to be the same compound (Supplementary Figure 1).

### Correlations between changing metabolites

**Figure 3:**
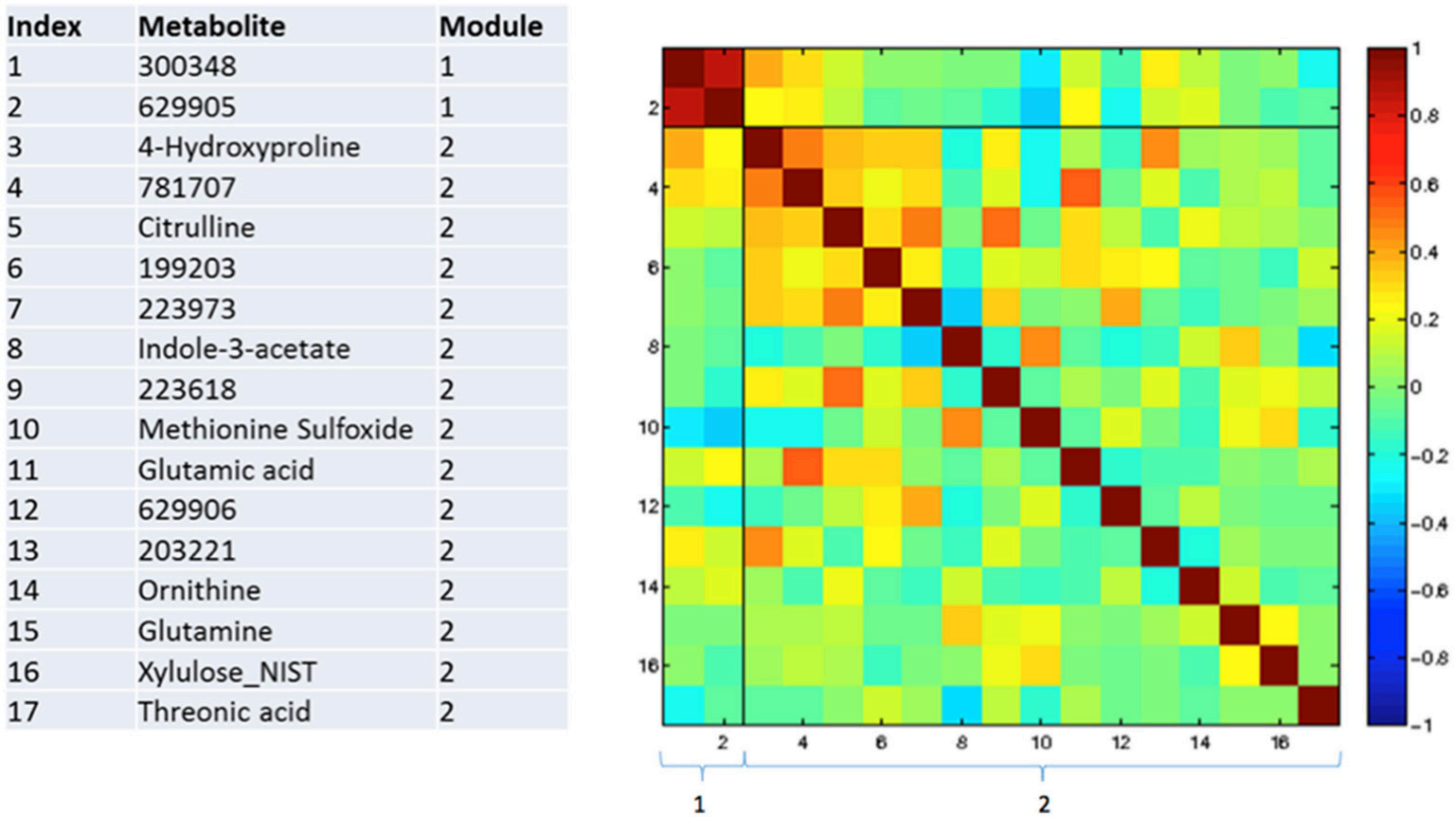
**Correlation and clustering between significant metabolites from time interval A to B**. The modules represent the grouping clusters of the metabolites. R, correlation coefficient (−1 < R < 1).

**Figure 4:**
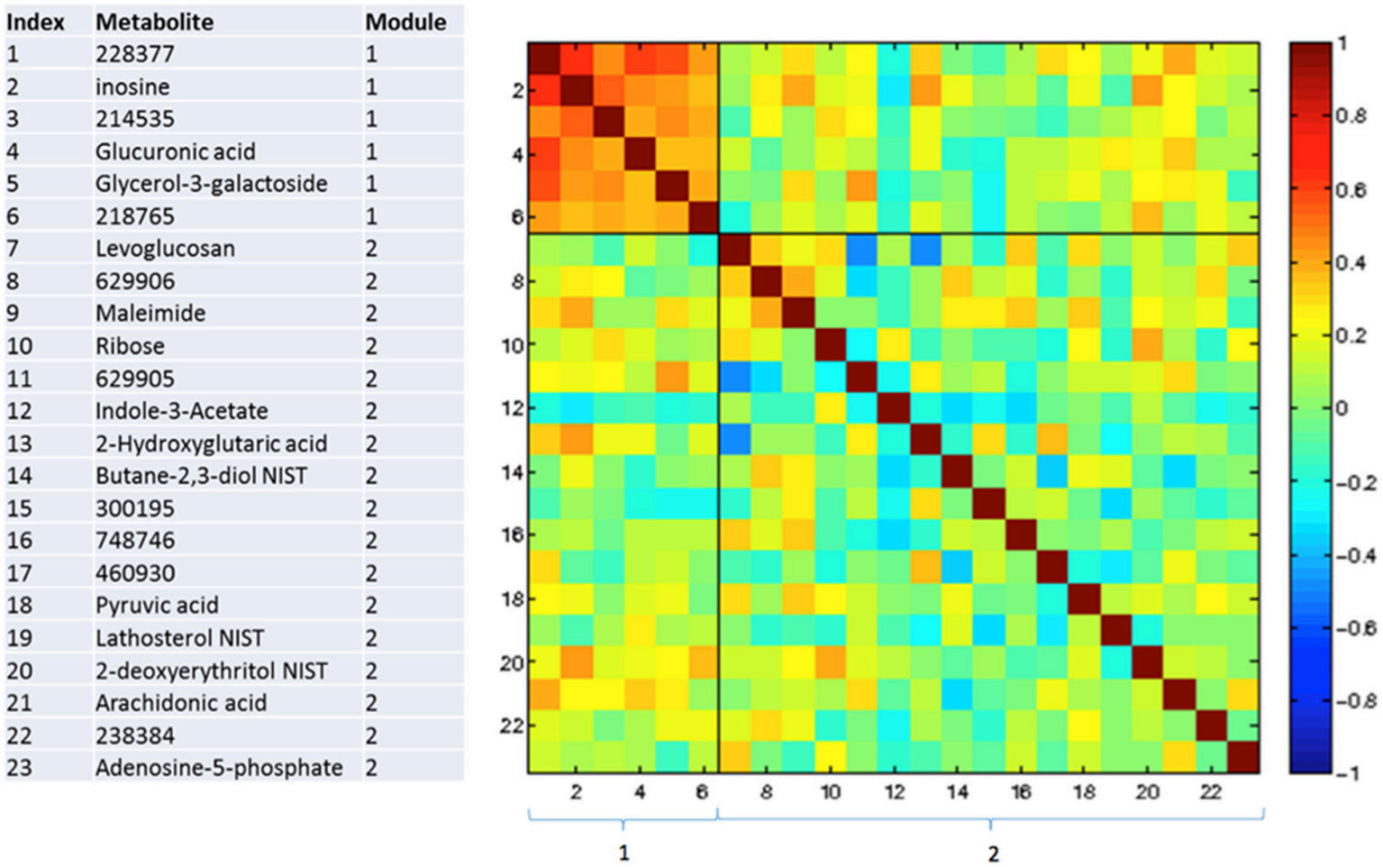
**Correlation and clustering between significant metabolites from time interval B to C**. The modules represent the grouping clusters of the metabolites. R, correlation coefficient (−1 < R < 1).

**Figure 5:**
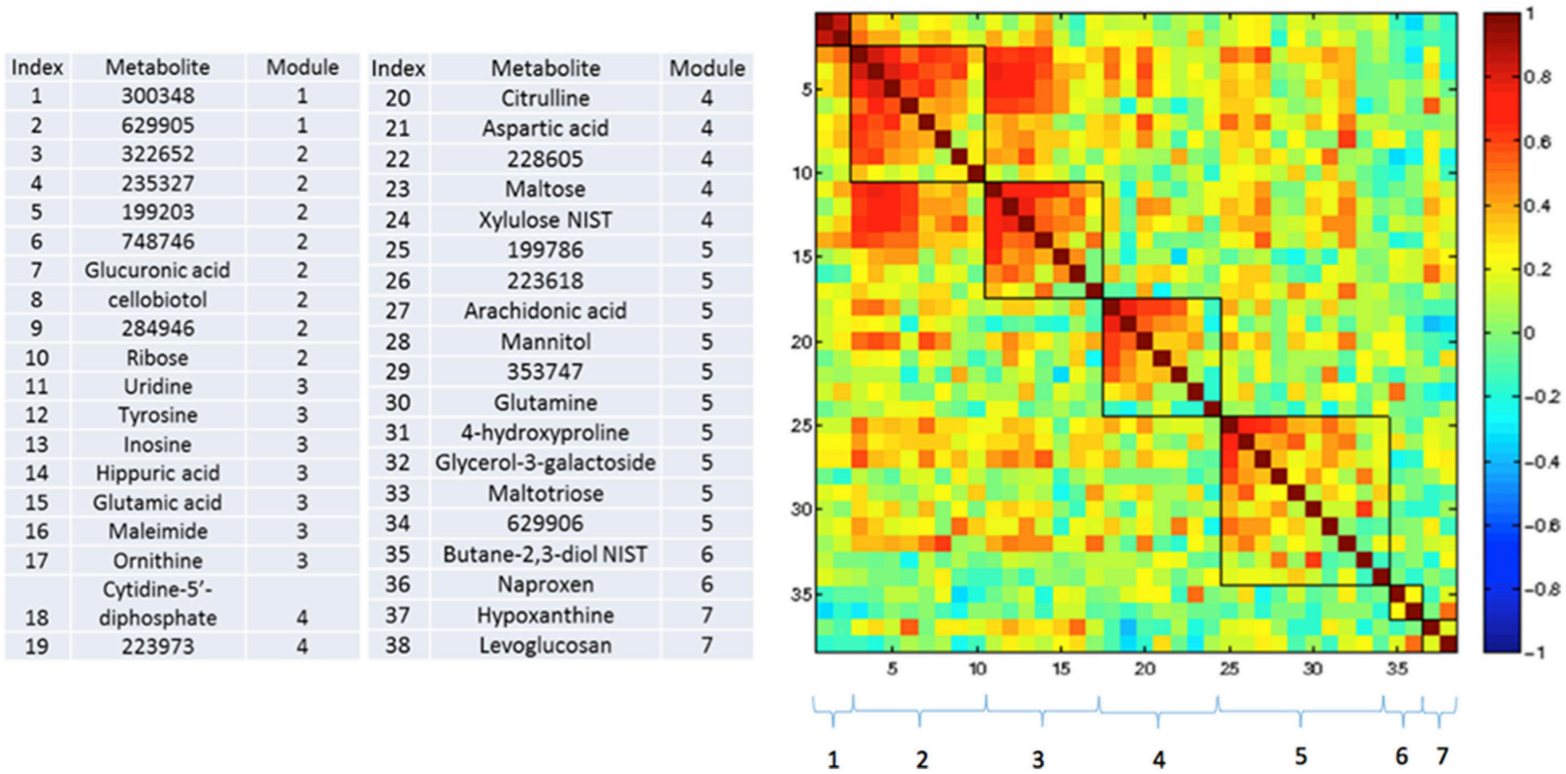
**Correlation and clustering between significant metabolites from time interval A to C**. The modules represent the grouping clusters of the metabolites. Shading represents the correlation coefficient (−1 < R < 1).

Figures 3–**5** show correlations between metabolites significantly altered due to metformin exposure for the given time interval (*q* < 0.2). The modules represent clusters determined using the MMC clustering algorithm (Stone and Ayroles, 2009) and show which metabolites are increasing or decreasing together. For time points A to B, only unknown metabolites 300348 and 629905 clustered together (Figure 3). For time points B to C, metabolites 228377, inosine, 214535, glucuronic acid, glycerol-3-galactoside, and 218765 clustered together (Figure 4). Lastly the pre-metformin (baseline) to peak metformin time point (A to C), produced seven modules with the module 1 containing unknown metabolites 300348 and 629905, consistent with time points A to B. Module 2 contained four unknown metabolites that clustered closely (322652, 235327, 199203, and 748746). Module 3 contained 7 metabolites, all of which have been structurally annotated (uridine, tyrosine, inosine, hippuric acid, glutamic acid, maleimide, and ornithine; Figure 5).

### Signature of association with peak metformin concentration

There were five metabolites significantly associated (*q* < 0.2) with maximum metformin concentration, Cmax, all for time point B comparisons. Of these five metabolites only one (Oxoproline) has been structurally identified. Results from the signature of metformin exposure are available in Supplementary File 1.

### Signature of association with metformin-mediated glucose response

Glucose AUC post-metformin was tested for significant correlations with metabolites measured at each time point (A, B, and C). No metabolites measured pre-metformin (baseline, time point A) were significantly correlated with glucose AUC post-metformin. Two metabolites at time point B (2-hydroxybutanoic acid, 2-deoxytetronic acid) were significantly and positively correlated with glucose AUC post-metformin (Table 4). For time point C, four metabolites were significantly correlated with glucose AUC post-metformin (fumaric acid, glycine, malic acid, and 2-hydroxybutanoic acid). Interestingly, 2-hydroxybutanoic acid at both time points B and C were positively correlated with glucose AUC post-metformin (Table 4). For the correlations of the change in metabolites for given time intervals, the time interval A to C was the only time interval analysis that had significant outcomes (*q* < 0.2). Overall, six metabolites that changed during this interval were significantly correlated with glucose AUC; all six of these metabolites were previously unknown (Table 4).

**Table 4:** **Metabolites significantly correlated with post-metformin glucose area under the plasma concentration time curve measured at various time intervals**.

**Time interval**	**Metabolite**	***R***	***q*-value**
B	2-hydroxybutanoic acid	0.607307	0.055967
B	2-deoxytetronic acid	0.546755	0.155866
C	Fumaric acid	−0.59113	0.104851
C	Glycine	−0.56567	0.104851
C	Malic acid	−0.55418	0.104851
C	2-hydroxybutanoic acid	0.538918	0.116422
A → C	^*^200476	0.554803	0.061408
A → C	^*^206556	0.571023	0.061408
A → C	^*^221574	0.580554	0.061408
A → C	^*^225398	0.595234	0.061408
A → C	^*^815932	0.559485	0.061408
A → C	^*^232017	0.508319	0.160358

* *Metabolite compounds yet to be structurally identified*.

### Pathway over-representation analysis

#### Signature of exposure

There were 33 known metabolites that were statistically significant during at least one time interval (A to B, B to C, or A to C) in the signature of metformin exposure analysis (*q* < 0.2) and 32 of these metabolites overlapped with at least one pathway in the HMDB database. Pathways were then tested to determine if any pathways in the HMDB database were enriched for these 32 metabolites. Significant pathways found in this analysis include, urea cycle, purine metabolism, ammonia recycling, arginine and proline metabolism, pyrimidine metabolism, amino sugar metabolism, histidine metabolism, glutamate metabolism, cysteine metabolism, and malate-aspartate shuttle pathways (*q* < 0.05; Table 5). The urea cycle pathway was significantly impacted based on the following seven metabolites: adenosine monophosphate, L-aspartic acid, citrulline, L-glutamic acid, L-glutamine, ornithine, and pyruvic acid, which overlapped with the 10 metabolites annotated in this pathway (KEGG ID: map00330). Metabolites adenosine monophosphate, L-glutamine, hypoxanthine, and inosine were also implicated in the purine metabolism pathway (*q* = 0.04; Table 5).

**Table 5:** **Results from the signature of exposure pathway analysis (*q* < 0.05)**.

**Pathway name**	**SMPDB ID^a^**	**KEGG ID^b^**	**Universe^c^**	**Pathway^d^**	**Group^e^**	**Overlapping^f^**	**Names of overlapping metabolites**	***q***
Urea cycle	SMP00059	map00330	137	10	32	7	Adenosine monophosphate, L-Aspartic acid, Citrulline, L-Glutamic acid, L-Glutamine, Ornithine, Pyruvic acid	0.004
Purine metabolism	SMP00050	map00230	137	6	32	4	Adenosine monophosphate, L-Glutamine, Hypoxanthine, Inosine	0.040
Ammonia recycling	SMP00009	map00910	137	9	32	5	Adenosine monophosphate, L-Aspartic acid, L- Glutamic acid, L-Glutamine, Pyruvic acid	0.040
Arginine and proline metabolism	SMP00020	map00330	137	7	32	4	L-Aspartic acid, Citrulline, L-Glutamic acid, Ornithine	0.040
Pyrimidine metabolism	SMP00046	map00240	137	5	32	3	CDP, L-Glutamine, Uridine	0.040
Amino sugar metabolism	SMP00045	map00520	137	5	32	3	L-Glutamic acid, L-Glutamine, Pyruvic acid	0.040
Histidine metabolism	SMP00044	map00340	137	3	32	2	Adenosine monophosphate, L-Glutamic acid	0.040
Glutamate metabolism	SMP00072	map00250	137	3	32	2	L-Glutamic acid, L-Glutamine	0.040
Cysteine metabolism	SMP00013	map00270	137	3	32	2	L-Glutamic acid, Pyruvic acid	0.040
Malate-aspartate shuttle	SMP00129		137	3	32	2	L-Aspartic acid, L-Glutamic acid	0.040

a Small Molecule Pathway Database ID;

b Kyoto Encyclopedia of Genes and Genomes ID;

c Number of metabolites in database that overlapped with metabolites tested using the metabolomics platform;

d Number of metabolites in pathway;

e Number of significant metabolites detected for at least one time point;

f *Number of significant metabolites detected that also overlapped with metabolites in the pathway*.

#### Signature of metformin response

Only three known metabolites (glycine, malic acid, and fumaric acid) were statistically significant in at least one time interval in the signature of metformin response analysis (*q* < 0.2) and overlapped with at least one pathway in the HMDB database (Supplementary Table 1). Overall, 14 pathways were significantly enriched for this set of metabolites (e.g., citric acid cycle, glutathione metabolism, alanine metabolism, gluconeogenesis, mitochondrial electron transport chain; Supplementary Table 1).

### Replication in mouse study

After intraperitoneal dosing of metformin (150 mg/kg for 7 days), significant decreases in ornithine, adenosine-5-phosphate, inosine, pyruvic acid, hypoxanthine, maltose, tyrosine, uridine, and aspartic acid were observed in serum samples from mice treated with metformin compared to mice treated with saline for the same length of time (*q* < 0.3; Supplementary Table 2). Indole-3-acetate and 4-hydroxyproline were elevated following metformin treatment, at all doses and 150 mg/kg, respectively, in the mice compared with mice treated with saline. Liver samples from the mouse model demonstrated significant decreases in levels of methionine sulfoxide, glutamine, inosine, maleimide, tyrosine, and uridine (*q* < 0.3) following metformin treatment at both 50 and 150 mg/kg doses (Supplementary Table 3). Additionally, liver samples from the mouse model demonstrated significant decreases in levels of ornithine, 2-hydroxyglutaric acid, and hypoxanthine at only the high dose of 150 mg/kg (*q* < 0.3) following metformin treatment (Supplementary Table 3).

## Discussion

Metformin is a commonly prescribed glucose-lowering medication; however, the underlying mechanism of action of metformin remains elusive. Here, we investigated the impact of metformin on a diverse set of metabolites using a non-targeted metabolomics approach in 33 subjects without a diagnosis of diabetes and of African-American ethnicity. The metabolites and biological pathways identified in our study provide information about signatures of metformin exposure and pharmacologic action. We first discuss our results in the context of exposure to metformin followed by its effects on glucose concentrations following an OGTT.

### Exposure to metformin

Differences in metabolite levels obtained at time points A and B, and B and C are both indicative of exposure to metformin. A to B differences reflect metabolites that increased or decreased 10 h after an oral dose of metformin; whereas differences in metabolite levels between time points B to C represent metabolites that increased or decreased 2 h after an oral dose of metformin. Unlike previous studies, which identified metabolite signature of metformin at steady state (Cai et al., 2009; Huo et al., 2009; Xu et al., 2015), our study highlights metabolite signatures after acute exposure. Of the metabolites with levels that changed in plasma after exposure to metformin (time points A to B), indole-3-acetate was notable because it was modulated in both the clinical and the mouse study. In the clinical study, indole-3-acetate levels were reduced 2 h after metformin administration (time point C compared with time point B) and increased 10 h after metformin administration (time point B compared with time point A). In mice, indole-3-acetate levels increased following metformin administration for 7 days at both 50 and 150 mg/kg doses. Indole-3-acetate is derived from the gut microbiota, and at time point C, estimated metformin concentrations in the intestine are about 25–50 mM (a 500–1000 mg dose in 250 mL intestinal fluid). These concentrations of metformin may have potent effects on the intestinal microbiome. In fact, several recent studies have shown that metformin has profound effects on the human gut microbiota (Lee and Ko, 2014; Zhang et al., 2015). The fact that indole-3-acetate levels were reduced in the 2 h sample but elevated in the 10 h sample of the clinical study as well as the mouse study may reflect differences in the effects of metformin on the formation, absorption and elimination kinetics of indole-3-acetate at various times. For example, high concentrations of metformin in the gut 2 h post-metformin dosing may affect the formation and the absorption of indole-3-acetate resulting in lower plasma levels; whereas at 10 h post-metformin dosing, metformin may inhibit the renal elimination of indole-3-acetate. Metformin has recently been shown to be an inhibitor of several transporters in the intestine and kidney (Chen et al., 2014; Liang et al., 2015). Interestingly, metabolites 629905 and 629906 were found to be significant when comparing all three different time points (A and B, B and C, A and C). Additional, spectral analysis suggests that these metabolites are likely to be the same compound (Supplementary Figure 1). However, it is noted that these two metabolites were only found in time point B and C, where the levels were higher in C compared to B and it is possible that these metabolites were part of the inert ingredients in the metformin tablet, as they were only observed after and not before metformin dosing.

Another notable metabolite that associated with exposure to metformin in both humans and mice was the metabolite, 4-hydroxyproline. In the clinical study, 4-hydroxyproline was significantly increased in the signature of metformin exposure for time points A to B, and A to C (*q* < 0.2). This effect was also observed in serum collected from mice receiving the 50 mg/kg dose and the 150 mg/kg dose (Supplementary Table 2). That is, increased 4-hydroxyproline levels were observed in mice receiving the 150 mg/kg relative to the 50 mg/kg dose of metformin. Although the mechanism by which metformin may result in increased serum levels of 4-hydroxyproline is not known, it is possible that metformin affects 4-hydroxyproline elimination or production, which is associated with collagen content in the body (Jenkins et al., 2003). Consistent with our results, significant changes in tissue and urine levels of 4-hydroxyproline have been noted in rodents treated with metformin (Kita et al., 2012; Lekshmi and Reddy, 2013).

Safety and efficacy biomarkers are topics of considerable interest in precision medicine for many diseases including diabetes (Pawlyk et al., 2014). In particular, the level of a biomarker (obtained before dosing) that predicts drug exposure, e.g., maximum plasma concentration of the drug, Cmax, may inform drug dosing (precision dosing). Unfortunately, the 11 baseline metabolites (obtained at time point A) that were associated with metformin Cmax were all of unknown structure, reflecting the current state of metabolomic research with many unknown metabolites. It would be of great interest to identify these metabolites and test them as biomarkers to inform metformin dosing and to gain insights into the mechanisms involved in metformin pharmacokinetics.

### Effects of metformin on oral glucose tolerance tests

Metformin elicits potent effects on glucose AUC after an OGTT, which is less apparent in non-diabetic subjects than in patients with T2D (Stocker et al., 2013). Metabolite levels that correlate positively with the change in glucose AUC upon metformin treatment may be considered as biomarkers of poor response to metformin. That is, relative to pre-metformin, no change or increased glucose levels indicate poor response whereas reduced glucose levels or AUC indicate a good response to metformin. In our study, 2-hydroxybutanoic acid levels (at both time points B and C) were positively correlated with glucose AUC post-metformin. Several studies have found that 2-hydroxybutanoic acid levels were associated with insulin resistance in non-diabetic subjects, and represent a potential early predictor of T2D and a biomarker for individuals at risk of developing T2D (Gall et al., 2010; Ferrannini et al., 2013; Muscelli et al., 2014). Our study showed that 2-hydroxybutanoic acid, at time point A, was positively correlated with fasting glucose before metformin administration (*r*^2^ = 0.14, *p* = 0.02), and glucose AUC before metformin administration (*r*^2^ = 0.20, *p* = 0.006), consistent with the metabolite being a biomarker of insulin resistance in non-diabetic subjects (Gall et al., 2010; Ferrannini et al., 2013). Furthermore, 2-hydroxybutanoic acid, at time points B and C, was positively correlated with glucose AUC after metformin administration (*r*^2^ = 0.31, *p* = 0.0004 and *r*^2^ = 0.21, *p* = 0.005) even when baseline glucose was subtracted from the glucose levels after the OGTT (data not shown). These data indicate that plasma levels of 2-hydroxybutanoic acid in addition to predicting poor glucose tolerance may be a predictor of poor response to metformin. These results need to be validated in patients with T2D.

### Pathway analyses

Pathway analysis revealed significant enrichment of metabolites in the urea cycle. This effect was driven largely by significant changes in ornithine, citrulline, and uridine, and adenosine-5-phosphate, which were all decreased upon exposure to metformin. Ornithine is decreased in the A to B time interval, and citrulline is decreased in the A to B, and A to C time intervals. Decreased ornithine was also observed in both liver and serum mouse samples. Our results are consistent with studies showing that ornithine and citrulline are significantly reduced in T2D patients treated with metformin (Irving et al., 2015; Xu et al., 2015). One potential reason for lower citrulline levels after metformin administration is its effects on mitochondrial complex I, the primary target of metformin (El-Mir et al., 2000; Owen et al., 2000). In particular, it is known that patients with mitochondrial deficiency (including patients with complex I deficiency) have significantly lower citrulline levels (Rabier et al., 1998; Atkuri et al., 2009). Ornithine and urea are produced by arginase 1 (ARG1) in the cytosol from arginine and water (Morris, 2002). Recently, metformin has been shown to reduce ARG1 activity (Bal et al., 2014), which is consistent with the reduced levels of ornithine observed in our study. Moreover, inosine was significantly decreased in plasma samples obtained at time points B and C after metformin exposure compared with samples obtained at time point A, and was also decreased in plasma samples obtained in mice treated with metformin. Altered inosine levels point toward changes in purine metabolism. Other pathways that were affected in our study include methionine and folic acid pathways, which have been shown to be altered in cancer cells (Cabreiro et al., 2013; Janzer et al., 2014). In the pathway analysis for the signature of metformin response, 14 pathways were significantly enriched for this set of metabolites (e.g., citric acid cycle, glutathione metabolism, alanine metabolism, gluconeogenesis, mitochondrial electron transport chain; Supplementary Table 1). Genes in many of these pathways have been implicated in metformin response in multiple studies (Chen et al., 2014; Pawlyk et al., 2014). However, each of these metabolites were enriched based on a single overlapping metabolite, and therefore, any conclusions drawn from this analysis should be interpreted cautiously.

It is important to consider that the results presented here were generated from non-diabetic subjects. Individuals with T2D diabetes would be expected to have significantly altered metabolic pathways. Therefore, the results presented here in non-diabetic subjects may be different from those observed in individuals with T2D. In addition, all of the subjects in this study were of African American ethnicity. African Americans have an increased risk of developing T2D than Caucasian individuals (Centers for Disease Control and Prevention, 2014); therefore, it is important to characterize the effects of metformin in this population, since they have an increased likelihood of receiving metformin over their lifetime. In addition, although we replicated results in the murine model, as with any model organism, species and metabolic differences may impact the outcome and limit the ability to translate results for human relevance. Additional research is needed using more targeted metabolomics platforms with in-depth coverage of the specific pathways identified in the present study. Further, identifying the structures of the “unidentified metabolites” will provide more information on metformin's effects. It is noteworthy, that six metabolites that were significantly associated with the OGTT response to metformin treatment (Table 4), and that 11 metabolites associated with metformin Cmax were all unidentified stressing the potential for new biomarkers that may provide the key for more targeted diabetes treatments in a highly susceptible population.

## Author contributions

DR and NO performed data analysis and wrote manuscript. XL and SY conducted experiments, performed analysis, and wrote manuscript. SS performed experiments. DC provided mechanistic insight and wrote manuscript. MM conducted pathway analysis. OF generated metabolomics data. AM designed analysis, KG and RK conceived and designed study. All authors read and approved the final manuscript.

### Conflict of interest statement

The authors declare that the research was conducted in the absence of any commercial or financial relationships that could be construed as a potential conflict of interest.
