# Supplementary material for: Pharmacometabolomic Assessment of Metformin in Non-diabetic, African Americans
Source: Front Pharmacol. 2016 Jun 14;7:135. doi: 10.3389/fphar.2016.00135 (PMC4906013; doi:10.3389/fphar.2016.00135)
Supplement: Supplementary file 1 [file DataSheet1.docx]

Supplementary Material

Pharmacometabolomic Assessment of Metformin in Healthy African American Subjects Implicates the Urea Cycle and Purine Metabolism

**Daniel M. Rotroff^1,2,¶^, Noffisat O. Oki^1,¶^, Xiaomin Liang^3^, Sook Wah Yee^3^, Sophie L. Stocker^3^, Daniel G. Corum^4^, Michele Meisner^2^, Oliver Fiehn^5,6^, Alison A. Motsinger-Reif^1,2^, Kathleen M. Giacomini^4,^*, Rima Kaddurah-Daouk^7,8,^***

^1^Bioinformatics Research Center, North Carolina State University, Raleigh, NC, USA

^2^Department of Statistics, North Carolina State University, Raleigh, NC, USA

^3^Department of Bioengineering and Therapeutic Sciences, University of California San Francisco, San Francisco, CA, USA

^4^Department of Regenerative Medicine and Cell Biology, Medical University of South Carolina, Charleston, SC, USA

^5^UC Davis Genome Center, University of California Davis, Davis, CA, USA

^6^ Department of Biochemistry, King Abdulaziz University, Jeddah, Saudi Arabia

^7^Department of Psychiatry and Behavioral Sciences, Duke University, Durham, NC, USA

^8^Duke Institute for Brain Sciences, Duke University, Durham, NC, USA

^¶^ Equal contribution

*** Correspondence:**

Rima Kaddurah-Daouk, Ph.D., Box 3903, 3552, Blue Zone, Duke South Durham, NC, 27710, USA

rima.kaddurahdaouk@duke.edu

Kathleen M. Giacomini, Ph.D., Department of Bioengineering and Therapeutic Sciences, University of California at San Francisco, 1550 4th Street, San Francisco, CA 94158, USA.

kathy.giacomini@ucsf.edu

# Supplementary Methods

## Signature of response of metabolites to metformin level

Univariate association for each metabolite to metformin level at the 3 time points was determined by linear regression using the open-source, statistical software, R(R Development Core Team, 2014). The analysis was performed using two variations of response: 1) metformin AUC, and 2) peak metformin concentration (C_max_). Covariates significantly associated with the phenotype were incorporated into the linear regression model to address potential issues of confounding. Gender, age, body mass index (BMI), weight, and height were tested for association with the each phenotype using a Pearson correlation coefficient = r>0.15. BMI was the only covariate that met this criterion and was subsequently included in the model described here:

$$Eq1. Y= \beta_{0}+\beta_{1}X_{1}+\beta_{2}X_{2}$$

Where, *Y*= AUC drug level or the peak drug level. *β*_0_ is the intercept, *β*_1…2_ is the corresponding regression coefficient. *X*_1_ is the metabolite measurement for the given time point, and *X*_2_ is BMI. The *p* values for *β*_1_ were corrected for multiple comparisons using an FDR approach and used to determine metabolite significance(Benjamini and Hochberg, 1995).

# Supplementary Figures


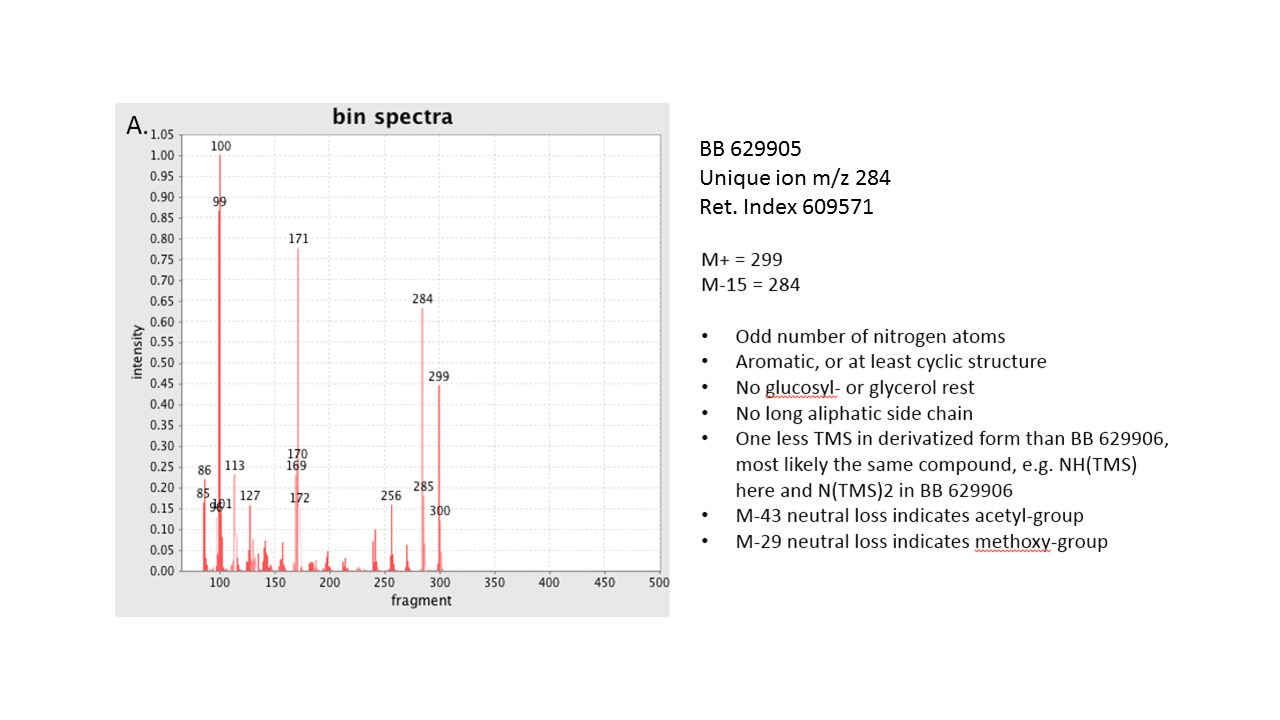


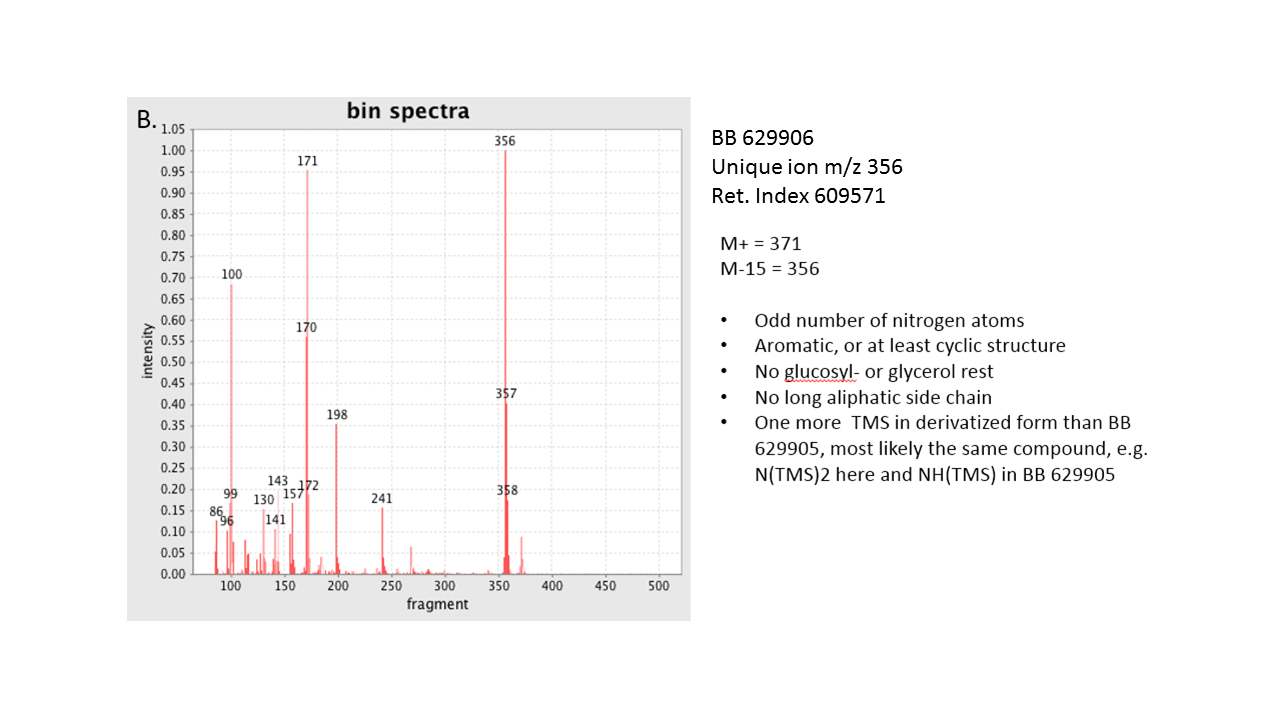


**Supplementary Figure 1.** A) Metabolite spectra and key features for unknown metabolite- 629905 and B) unknown metabolite-629906. Comparisons of spectra and key features suggest that these two unknown metabolites are likely to be the same compound.

# Supplementary Tables

**Supplementary Table 1.** Results from pathway analysis of metabolite changes with glucose AUC (q<0.05)

| Number of Metabolites in: | | | | | | | | |
| --- | --- | --- | --- | --- | --- | --- | --- | --- |
| **Pathway Name** | **SMPDB ID^a^** | **KEGG ID^b^** | **Universe^d^** | **Pathway^e^** | **Group^f^** | **Overlapping^g^** | **Names of Overlapping metabolites** | **q** |
| Methionine Metabolism | SMP00033 | map00270 | 1061 | 24 | 3 | 1 | Glycine | 0.088 |
| Ammonia Recycling | SMP00009 | map00910 | 1061 | 24 | 3 | 1 | Glycine | 0.088 |
| Urea Cycle | SMP00059 | map00330 | 1061 | 20 | 3 | 1 | Fumaric acid | 0.088 |
| Arginine and Proline Metabolism | SMP00020 | map00330 | 1061 | 26 | 3 | 1 | Fumaric acid | 0.088 |
| Phenylalanine and Tyrosine Metabolism | SMP00008 | map00360 | 1061 | 13 | 3 | 1 | Fumaric acid | 0.088 |
| Aspartate Metabolism | SMP00067 | map00250 | 1061 | 12 | 3 | 1 | Fumaric acid | 0.088 |
| Porphyrin Metabolism | SMP00024 | map00860 | 1061 | 22 | 3 | 1 | Glycine | 0.088 |
| Carnitine Synthesis | SMP00465 |  | 1061 | 17 | 3 | 1 | Glycine | 0.088 |
| Citric Acid Cycle | SMP00057 | map00020 | 1061 | 23 | 3 | 1 | Fumaric acid | 0.088 |
| Glutathione Metabolism | SMP00015 | map00480 | 1061 | 10 | 3 | 1 | Glycine | 0.088 |
| Alanine Metabolism | SMP00055 | map00250 | 1061 | 20 | 3 | 1 | Glycine | 0.088 |
| Gluconeogenesis | SMP00128 | map00010 | 1061 | 27 | 3 | 1 | Malic acid | 0.088 |
| Mitochondrial Electron Transport Chain | SMP00355 | map00190 | 1061 | 15 | 3 | 1 | Fumaric acid | 0.088 |
| Malate-Aspartate Shuttle | SMP00129 |  | 1061 | 8 | 3 | 1 | Malic acid | 0.088 |

^a^Small Molecule Pathway Database ID; ^b^Kyoto Encyclopedia of Genes and Genomes ID; ^c^Pathway was tested using baseline metabolite levels associated with change in HDBP (Baseline Response), changing metabolites associated with change in HDBP (Change Response), or baseline metabolites associated with drug treatment (Exposure); ^d^Number of metabolites in database that overlapped with metabolites tested using the metabolomics platform; ^e^Number of metabolites included in pathway; ^f^Number of significant metabolites detected; ^g^Number of significant metabolites detected that also overlapped with metabolites in the pathway.

**Supplementary Table 2.** Mean serum metabolites levels in mice treated with saline, metformin 50mg/kg and metformin 150mg/kg (N=6 per group) and the q-value between each treatment group. These serum metabolites were significant in human studies shown in Table 1 to Table 3. Values shaded in green were significantly increased, whereas, values shaded in red were significantly decreased (q < 0.3).

| **Metabolite** | **Mean saline** | **mean metformin 50mg/kg** | **mean metformin 150mg/kg** | **saline vs 50mg/kg** | **saline vs 150mg/kg** | **50mg/kg vs 150mg/kg** |
| --- | --- | --- | --- | --- | --- | --- |
| Citrulline | 976 | 1121 | 814 | 0.846 | 0.289 | 0.422 |
| Ornithine | 10771 | 10510 | 6547 | 0.970 | 0.162 | 0.267 |
| 4-hydroxyproline | 381 | 302 | 419 | 0.695 | 0.636 | 0.267 |
| Methionine sulfoxide | 1548 | 784 | 1032 | 0.546 | 0.364 | 0.544 |
| Glutamic acid | 1479 | 2217 | 1110 | 0.695 | 0.482 | 0.267 |
| Indole-3-acetate | 1790 | 2179 | 2614 | 0.273 | 0.035 | 0.267 |
| Glutamine | 9205 | 11052 | 8446 | 0.846 | 0.709 | 0.504 |
| Threonic acid | 17762 | 18848 | 20477 | 0.737 | 0.364 | 0.544 |
| Glycerol-3-galactoside | 230 | 141 | 141 | 0.695 | 0.398 | 0.990 |
| Adenosine-5-phosphate | 1285 | 1784 | 532 | 0.695 | 0.139 | 0.021 |
| Inosine | 277 | 307 | 181 | 0.846 | 0.021 | 0.267 |
| 2- Hydroxyglutaric acid | 469 | 477 | 433 | 0.970 | 0.661 | 0.530 |
| Arachidonic acid | 1959 | 2088 | 1819 | 0.846 | 0.661 | 0.366 |
| Maleimide | 366 | 304 | 323 | 0.695 | 0.508 | 0.737 |
| Pyruvic acid | 1469 | 857 | 562 | 0.695 | 0.009 | 0.544 |
| Hypoxanthine | 137 | 152 | 48 | 0.970 | 0.289 | 0.292 |
| Maltose | 132 | 133 | 103 | 0.982 | 0.286 | 0.422 |
| Tyrosine | 19761 | 19198 | 17138 | 0.846 | 0.162 | 0.267 |
| Maltotriose | 27 | 26 | 23 | 0.970 | 0.508 | 0.587 |
| Uridine | 1160 | 1137 | 799 | 0.970 | 0.162 | 0.298 |
| Aspartic acid | 857 | 1331 | 545 | 0.695 | 0.100 | 0.267 |

**Supplementary Table 3**. Mean metabolites levels in the liver of the mice treated with saline, metformin 50mg/kg and metformin 150mg/kg (N=6 per group) and the q-value between each treatment group. These metabolites were significant in human studies shown in Table 1 to Table 3. Values shaded in green were significantly increased, whereas, values shaded in red were significantly decreased (q < 0.3).

| **Metabolite** | **Mean saline** | **mean metformin 50mg/kg** | **mean metformin 150mg/kg** | **saline vs 50mg/kg** | **saline vs 150mg/kg** | **50mg/kg vs 150mg/kg** |
| --- | --- | --- | --- | --- | --- | --- |
| Citrulline | 383 | 340 | 311 | 0.730 | 0.802 | 0.749 |
| Ornithine | 13191 | 8783 | 5134 | 0.400 | 0.115 | 0.146 |
| 4-hydroxyproline | 1177 | 257 | 375 | 0.518 | 0.802 | 0.359 |
| Methionine sulfoxide | 953 | 704 | 654 | 0.104 | 0.053 | 0.587 |
| Glutamic acid | 31669 | 22156 | 32905 | 0.207 | 0.910 | 0.146 |
| Glutamine | 6905 | 3828 | 1906 | 0.182 | 0.053 | 0.040 |
| Threonic acid | 527 | 629 | 512 | 0.136 | 0.910 | 0.277 |
| Glycerol-3-galactoside | 482 | 278 | 478 | 0.104 | 0.959 | 0.080 |
| Adenosine-5-phosphate | 4210 | 4213 | 4092 | 0.993 | 0.910 | 0.752 |
| Inosine | 30779 | 23406 | 22274 | 0.104 | 0.053 | 0.749 |
| 2-Hydroxyglutaric acid | 468 | 437 | 337 | 0.720 | 0.158 | 0.146 |
| Arachidonic acid | 3851 | 4964 | 4021 | 0.182 | 0.910 | 0.277 |
| Maleimide | 695 | 500 | 477 | 0.117 | 0.071 | 0.752 |
| Pyruvic acid | 432 | 461 | 599 | 0.869 | 0.330 | 0.323 |
| Hypoxanthine | 14449 | 13256 | 10317 | 0.518 | 0.053 | 0.146 |
| Maltose | 41274 | 6113 | 31480 | 0.104 | 0.802 | 0.080 |
| Tyrosine | 15155 | 12356 | 11063 | 0.128 | 0.053 | 0.323 |
| Maltotriose | 1987 | 253 | 2304 | 0.117 | 0.910 | 0.146 |
| Uridine | 10568 | 8476 | 7876 | 0.104 | 0.053 | 0.675 |
| Aspartic acid | 17782 | 16411 | 17229 | 0.518 | 0.910 | 0.749 |

# Supplementary Files

Supplemental_File_1.xlsx: Information on study subjects.

Supplemental_File_2.xlsx: Clinical variables from study subjects including drug level and glucose levels.

Supplemental_File_3.xlsx: Metabolomics data.

Supplemental_File_4.xlsx: Univariate metabolite associations.

Supplemental_File_5.xlsx: Mouse metabolomics data.

# References

Benjamini, Y., and Hochberg, Y. (1995). Controlling the false discovery rate: a practical and powerful approach to multiple testing. *J. R. Stat. Soc. Ser. B Methodol.*, 289–300.

R Development Core Team (2014). R: A language and environment for statistical computing. R Foundation for Statistical Computing, Vienna, Austria. ISBN 3-900051-07-0, URL http://www.R-project.org/. Available at: http://www.R-project.org.
